# Pseudolumen Size and Perimeter in Prostate Cancer: Correlation with Patient Outcome

**DOI:** 10.1155/2011/693853

**Published:** 2011-07-14

**Authors:** Kenneth A. Iczkowski, Kathleen C. Torkko, Gregory R. Kotnis, R. Storey Wilson, Wei Huang, Thomas M. Wheeler, Andrea M. Abeyta, M. Scott Lucia

**Affiliations:** ^1^Department of Pathology, University of Colorado Denver School of Medicine, Aurora, CO 80045, USA; ^2^2100 Marble Cliff Office Park, Suite A, Columbus, OH 43215, USA; ^3^Department of Pathology and Laboratory Medicine, University of Wisconsin, Madison, WI 53792, USA; ^4^Department of Pathology and Immunology, Baylor College of Medicine, Houston, TX 77030, USA

## Abstract

We demonstrated in 2011 that 61% of men with postoperative PSA failure had some cribriform pattern of prostate cancer, versus 16% of nonfailures (OR = 5.89, *P* < .0001). That study used digitized radical prostatectomy slides from 153 men, 76 failures (≥0.2 ng/mL) matched to 77 nonfailures. The current study's hypothesis: pseudolumen size and shape variability could stratify outcome within histologic patterns (single separate acini, separate acini with undulating lumens, fused small acini, papillary, cribriform). Pseudolumens were filled digitally on image captures from previously annotated specimens. Among all 5 patterns, pseudolumen spaces averaged smaller in failures than nonfailures. After multivariate analysis controlling for stage, age, margin, cancer amount, prostate volume, and presence of individual cells (grade 5), this retained significance only for the undulating-lumens and papillary patterns. In undulating-lumens pattern, PSA failures had smaller mean pseudolumen space sizes (*P* = .03) but larger perimeters (*P* = .04), implying more pseudolumen irregularity. In papillary pattern, the number of pseudolumen spaces was higher in failures (*P* = .015), space size was smaller (*P* = .11), perimeters were smaller (*P* = .04), and perimeter/size ratio was higher (*P* = .02). In conclusion, digitally measured pseudolumen size and shape may associate with outcome.

## 1. Introduction

In 1966, a 5-tier prostate cancer grading system that relies entirely on architectural features was devised by Donald Gleason, who correlated patient outcome with the histologic patterns in 270 Veterans Administration patients [1]. The Gleason system is now recognized to predict pathologic stage and guides treatment choice. A few refinements have been introduced to the grading system, some by Gleason himself [2, 3]. Grades of 1 and 2, the lowest grades, have fallen into clinical irrelevance [4, 5], and grade 5 is fairly rare, and thus most cancers are grade 3, grade 4, or a combination of the two, creating three usual diagnostic bins. Gleason later designated a large acinar, undulating pattern of separate acini as pattern 3A and cribriform/papillary carcinoma as pattern 3C [3]. Cribriform cancer in 2000 was still deemed grade 3 [6], but the grading of cribriform cancer later became controversial [3, 7–10]. 

Within any pattern of cancer, there is a continuum of size, shape, and spacing of pseudolumens. The current work aims to discover whether these relative differences in pseudolumen morphology for any histologic pattern can substratify its association with outcome. We addressed this question by capturing images within a set of 153 previously scanned and annotated specimens [11], then annotating and analyzing the number, size, and shape of pseudolumens. Moreover, in our recent study, within the continuum of histologic patterns considered grade 4, the cribriform pattern was associated with a distinctly adverse outcome [11]. In the prostate cancer population that was studied, the frequency of any cribriform cancer was 38% of specimens: a higher frequency than the frequency of 23% for individual cells, the prototypical Gleason grade 5. Yet, PSA failure was even more strongly associated with presence of cribriform pattern than with presence of individual cell pattern; thus, the current study paid special attention to cribriform pattern.

## 2. Materials and Methods

The study used the set of slides from 153 cases of prostate cancer that we published previously [11]. Men chosen for the study came from 3 medical centers: the University of Colorado Denver Hospital (*n* = 44), University of Wisconsin Health System (*n* = 60), and Methodist Hospital, Houston (*n* = 49). Exclusion criteria were a history of receiving cryotherapy, radiotherapy, or androgen deprivation before failure. All men had postoperative drops in PSA to undetectable. Most had Gleason score 7 cancer (95% in the 6–8 range). Biochemical failure was considered as a rise to ≥0.2 ng/mL, without evidence of later, lower measurements that would invalidate the 0.2. All but 2 nonfailures had at least 2 years' followup; 2 had between 1 and 2 years' followup. At all contributing sites, prostates were completely sampled at 4–6 mm intervals. 

### 2.1. Histologic Pattern and Pseudolumen Annotation

Patients' entire prostatectomy slide sets had been re-reviewed. Those slides containing cancer (average per case, 8.0 ± 4.3) were digitally scanned as virtual slides using an Aperio ScanScope XT at a resolution of 0.50 um/pixel (Aperio Technologies, Vista, Calif) [11]. Using Image Scope software, all foci of 6 histologic patterns were previously manually annotated in a nonoverlapping manner, using a different color for each pattern, denoted as follow: (1) S: single, separate small acini like 3B pattern [3]; (2) U: undulated, stellate, or branching medium acini, like 3A pattern [3]; (3) F: fused, ragged small acini including those with mucin; (4) P: (micro)papillary consisting of medium to large spaces with either stromal cores or strands of cells with one or more cell layers bridging across the acinus, with intervening slit-like spaces; (5) C: cribriform, with medium to large acinar spaces having punched-out lumens (inclusive of the glomeruloid pattern [12]); (6) I: individual infiltrating or sheet-like cells lacking lumen formation. For each specimen, a representative 1500 × 1000 pixel section was captured from each annotated pattern (excluding I pattern, since it lacks lumens). All pseudolumen spaces in each image were further annotated using Photoshop. Annotated pseudolumen spaces were then analyzed for several different morphometric criteria using a custom written ImageJ plugin. Measurements for pseudolumen object frequency, area, circularity, perimeter, and maximum and minimum feret diameter were recorded and formatted for subsequent analysis.

Subsequent analysis was focused on the 48 specimens with cribriform pattern, of which 37 had PSA failure, compared to 9 without failure. For each, a second 1500 × 1000 pixel ImageScope snapshot was taken from cribriform areas, using cribriform foci from different slides than previous or different foci from the same slide, wherever possible (*n* = 43). In 5 cases the small size of the cribriform area precluded capturing two snapshots without overlap, so analysis was based on one snapshot. Pseudolumen space annotation and analysis were carried out as above.

### 2.2. Statistical Analysis

For each patient, biochemical failure status was recorded. To determine whether failure was related to any measurement for the patterns or to preoperative serum PSA, pathologic stage, grade, and margin status (but not Gleason score, owing to collinearity with the measured data), univariate and multivariate analyses were used. Only multivariate analysis results are displayed. 

Depending on the distribution of the underlying data, either parametric or nonparametric tests were used to determine the associations of biochemical failure with clinical and pathologic parameters. Logistic regression analysis was used to test associations with failure while adjusting for potential confounding variables. All tests were two-sided, and significance level was set at *P* < .05. All analyses used SAS version 9.2 (SAS Institute, Cary, NC).

## 3. Results

The cases and controls were matched on length of follow-up, age at surgery, stage, and grade. Clinicopathologic data on their specimens, from which 1,100 slides were digitized and annotated, proved that a successful match was made on age and length of followup [11]. With our limited number of possible controls, however, stage and grade were not completely matched. To account for any mismatching, we adjusted our multivariate models for these potential confounders. Because we did match, or attempted to match on age, stage, grade, and length of followup, the independent relationship of these variables to PSA failure could not be assessed, as the distribution of these variables in controls did not represent a random sample. 

**Figure 1:**
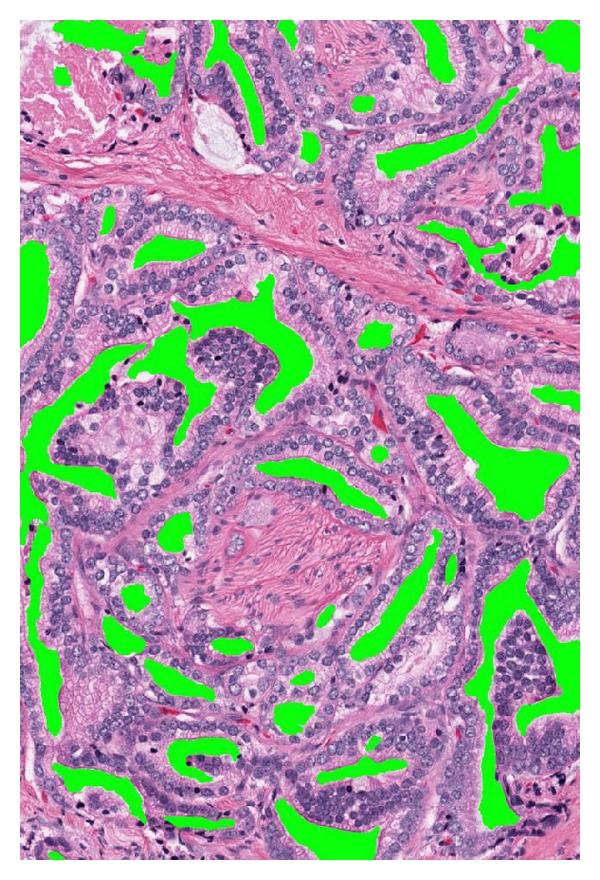
Image capture of undulating lumens grade 3 pattern, 1500 × 1000 pixels, after pseudolumen annotation. Lumens are very irregular; there is perineural invasion. Patient had PSA failure (100x).

**Figure 2:**
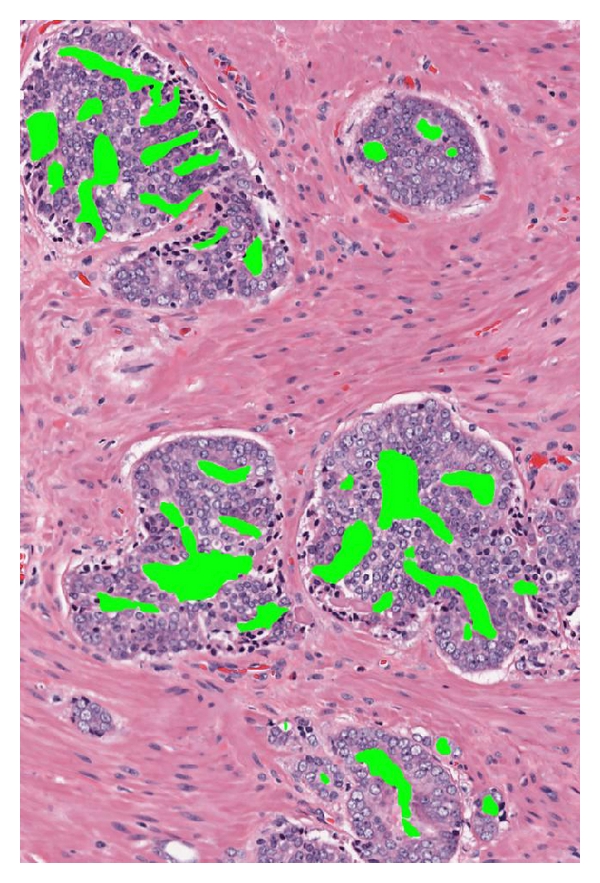
Image capture of true papillary grade 4 pattern, 1500 × 1000 pixels, after pseudolumen annotation. Note bridging across gland space. Patient had PSA failure (100x).

**Figure 3:**
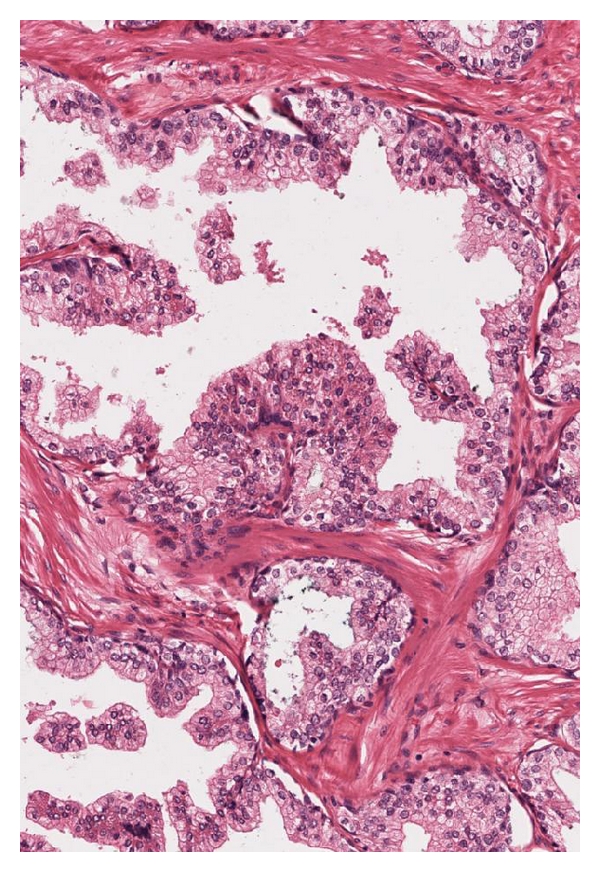
Benign prostatic acini can have undulating, or papillary lumens have lumens that are large and open, usually without bridging across the gland space except in the central zone. Pseudolumen spaces in cancer show various degrees of deviation from normal (100x).

By our definition of PSA failure, the 153 total patients comprised 76 (49.7%) with PSA failure and 77 (50.3%) without failure. Five had known metastases, and 6 had prostatic fossa recurrence. There were 5 deaths from prostate cancer (3.3%) and 2 from other causes in the study. As expected, patient age, follow-up days, and prostate volume were similar with respect to PSA failure status. 105 (68.6%) specimens were Gleason ≥7, 67 (43.8%) were stage T3, and 51 (33.3%) specimens had positive margins. Table 1 demonstrates that there were significant differences between the failures and nonfailures for many measurements for single separate acini, separate acini with undulating lumens, fused small acini, and papillary acini. As a general trend across all patterns, the mean pseudolumen space size was smaller in failures than in nonfailures. These differences were adjusted by multivariate analysis for preoperative serum PSA, pathologic stage, grade, and margin status, and the presence of the individual cell pattern (I) which by definition had to be a categorical variable due to having no annotatable lumens. Significance for odds ratios of failure persisted for the undulating-lumens and papillary patterns. In the undulating-lumens pattern (Figure 1), PSA failures had smaller mean pseudolumen space sizes (*P* = .03) but larger pseudolumen perimeters (*P* = .04), implying more irregularity of the spaces. In the true papillary pattern (Figure 2), the number of pseudolumen spaces was higher in failures (*P* = .015), the size of spaces was smaller (*P* = .11), the perimeter of spaces was smaller (*P* = .04), and the perimeter/size ratio was higher (*P* = .02). The latter result again implies more irregularity of the spaces. These both stand in contrast to benign acini, which have large, open lumens (Figure 3).

For the cribriform pattern, some correlations based on one image capture per specimen were significant, and since our prior work had shown that the cribriform pattern carries the most adverse prognosis of all patterns examined [11], we chose to expand the data by capturing a second set of nonoverlapping cribriform cancer images from a different slide where possible. Notably, no specimen had any necrosis within the cribriform component and none of the cribriform areas fit the criteria for ductal carcinoma morphology [13–15]. In Table 2, the median of pseudolumen space size was smaller (133.5) in the failures compared to the failures (96.1) (*P* = .048). The median perimeter of pseudolumens was 167.5 in the failures versus 206.2 in nonfailures (*P* = .070). However, the perimeter/size ratio, describing the degree of lumen contour irregularity, was 1.75 in the failures, higher than the 1.41 in nonfailures (*P* = .074). 

The individual cell (I)/Gleason 5 pattern was seen in 35 patients (23%) and in 13 (27%) of 48 specimens with cribriform pattern, all but one of which belonged to a man with PSA failure. A previous study had shown that C pattern frequently coexisted with I pattern, and these were multiplicative with regard to odds ratio for failure and probably minimized the independent associative value for the fairly rare I pattern [11]. Multivariate analysis, as above, considered the presence of I pattern. Size of pseudolumens retained significance (*P* = .040), and perimeter had borderline significance (*P* = .058). An increased perimeter/size ratio gave a high OR for failure, but this missed significance.

## 4. Discussion

Large acinar (LA) prostate cancer, comprising the cribriform (C) and papillary (P) patterns, is associated with higher PSA failure odds ratios than other high-grade patterns [11]. Here we have shown, firstly, that there was a trend toward smaller lumen sizes in all 5 patterns. After adjusting for other clinicopathologic findings by multivariate analysis, this held significance for separate, medium-sized glands with undulating lumens and for the P pattern. For the undulating-lumens pattern, larger perimeter of the gland spaces was noted in men with PSA failure, suggesting that a greater complexity of infoldings in this Gleason grade 3 pattern correlates with advanced tumor development. Moreover, within the grade 4, P pattern, there were significant trends for increased number, smaller size, and greater irregularity (perimeter/size ratio) of pseudolumen spaces to be associated with PSA failure. This suggests that, as its epithelium assumes a more complex, nearly solid pattern, there is more advanced tumor development. Finally, the number of men with C pattern was too small of a sample set, with only 9 of 48 men without failure, precluding much statistical significance for its pseudolumen findings, but further study is warranted. 

Cribriform and papillary (LA) cancers, as recently as 2000, were placed under grade 3 by a consensus statement of the College of American Pathologists [6]. Subsequent evidence for an elevated biologic potential of cribriform cancer came from Kronz et al., who, in their biopsy study of “atypical cribriform lesions,” found that 55% of patients had cancer on repeat biopsy. Also, of 10 patients with subsequent carcinoma, 6 had a component of Gleason pattern 4 [9]. The International Society of Urological Pathology (ISUP) grading consensus conference of 2005 judged that most cribriform structures were grade 4, but those that were rounded and of comparable size to benign acini could be graded as grade 3 [7]. Cribriform pattern (small or large) was present in 60.5% of men with PSA failure but only 15.6% of matched men without failure, resulting in an odds ratio (OR) for PSA failure of 5.89, higher than for any other pattern. The adverse implications of cribriform pattern presence did not depend on whether the cribriform area was large and sprawling, or small, round, and circumscribed. Papillary (P) pattern, the other LA pattern, had a somewhat lower but significant 2.155 odds ratio for PSA failure [11]. Strikingly, among 17 cases with a preponderance of LA (cribriform and papillary) patterns amounting to 1/3 of cancer area, the odds ratio for failure reached 10.80. The ability of pseudolumen measurements to further stratify outcome within LA cancer warrants further investigation. 

The only other digital analysis of the correlation of morphometric imaging features with clinical failure was that of Donovan et al. from Aureon Laboratories [16]. That study used a model that incorporated the spacing between epithelial tumor cells and the ratio of epithelial tumor cell area to total tumor area, in grade 3 cancer in prostatic needle biopsies. Combined with these morphologic data were quantitative multiplex immunofluorescence results and three clinicopathologic features. The current study, in contrast, assessed five pseudolumen-forming patterns identifiable in grades 3 and 4 cancer and indirectly approximated the percent of epithelium present by measuring the proportion of pseudolumen area in standard size images, as well as the shapes of those spaces. An asset of this approach is cost-effective outcome prediction without the need for immunofluorescence. A limitation was the inability to mark epithelium differently from stroma (which might be done in future studies using a reticulum stain, a technique that may have sharpened the discrimination of the observed associations).

Because of several other limitations of the current study, validation studies using unselected cases will be needed before results are applied to the population of prostate cancer patients diagnosed on biopsy. The first limitation was that PSA failures were enriched. Since failure is a rarer outcome than nonfailure, it was necessary to overrepresent failures in the matching process to try for statistical significance. Matching the nonfailures for stage, grade, and margins also skewed the nonfailure population to those with more adverse features. Second, the use of prostatectomy specimens was selected for those men who chose surgery and who probably had more aggressive cancer than average. This too was necessary, in order to quantify the entire cancer in the gland without the sampling error inherent in biopsies. A prospective study on biopsy material would be needed to represent the entire spectrum of men with cancer, some of whom elect watchful waiting, hormone ablation, radiation, or cryotherapy. Hence, this study's findings apply best to men who are surgical candidates. Finally, using PSA failure is a valid endpoint, but less definite than death from cancer; however, the study would have had to include many more men to amass a sufficient number of deaths.

## 5. Conclusion

Morphometry of the pseudolumens of certain acinar patterns—separate acini with undulating lumens, papillary, and cribriform—may correlate with biochemical failure in prostate cancer. Some limitations of this study render these results preliminary, but future studies should use an unselected population to examine the relationship of lumen size, shape, and spacing within annotated patterns to outcome.

## Figures and Tables

**Table 1:** Pseudolumen measurements according to pattern, other than cribriform.

Histologic pattern	Nonfailures	Failures	Median for nonfailure	Median for failure	*P* value, Wilcoxon Rank Sum	Odds ratio, adjusted*	*P* value for odds ratio
Single separate acini	*n* = 80	*n* = 68					
No. of pseudolumen spaces			39.5	42.9	.49	0.997	.79
Size of spaces based on mean, *μ*m^2^			160	130	.03	0.998	.16
Perimeter of spaces, *μ*m			233	204	.004	0.996	.09
Perimeter/size ratio			1.49	1.58	.17	1.29	.49
Undulating-lumen separate acini	*n* = 61	*n* = 50					
No. of pseudolumen spaces			34.0	30.5	.23	0.971	.06
Size of spaces based on mean, *μ*m^2^			342.9	314.5	.77	1.001	**.03**
Perimeter of spaces, *μ*m			363	416	.69	1.002	**.04**
Perimeter/size ratio			1.19	1.21	.89	0.576	.39
Fused small acini:	*n* = 62	*n* = 62					
No. of pseudolumen spaces			47.5	46.5	.75	0.975	.98
Size of spaces based on mean, *μ*m^2^			66.5	49.1	.002	0.066	.07
Perimeter of spaces, *μ*m			147	117	.002	0.153	.15
Perimeter/size ratio			2.21	2.46	.003	0.059	.06
True papillary acini	*n* = 31	*n* = 43					
No. of pseudolumen spaces			28.0	43.0	.008	1.05	**.015**
Size of spaces based on mean, *μ*m^2^			355.7	190.8	.025	0.99	.11
Perimeter of spaces, *μ*m			382	262	.023	0.997	**.04**
Perimeter/size ratio			1.16	1.46	.061	4.77	**.02**

*By multivariate analysis adjusting for preoperative serum PSA, pathologic stage, grade, margin status, and the presence of the individual cell pattern.

**Table 2:** Pseudolumen measurements for the cribriform pattern.

Cribriform acini	Median for nonfailure, *n* = 9	Median for failure, *n* = 39	*P* value, Wilcoxon Rank Sum	Odds ratio, adjusted*	*P* value for odds ratio
No. of pseudolumen spaces	37	48	.552	1.022	.394
Size of spaces based on mean, *μ*m^2^	133.5	96.0	.125	0.987	**.048**
Perimeter of spaces, *μ*m	206.2	167.5	.125	0.987	.070
Perimeter/size ratio	1.41	1.75	.178	7.076	.074

*By multivariate analysis adjusting for preoperative serum PSA, pathologic stage, grade, margin status, and the presence of the individual cell pattern.
